# Neurotherapeutic potential of intranasal administration of human breast milk

**DOI:** 10.1038/s41390-023-02759-z

**Published:** 2023-07-26

**Authors:** Atul Malhotra

**Affiliations:** 1 Department of Paediatrics, Monash University, Melbourne, VIC Australia; 2 Monash Newborn, Monash Children’s Hospital, Melbourne, VIC Australia; 3 The Ritchie Centre, Hudson Institute of Medical Research, Melbourne, VIC Australia

Benefits of human breast milk for the newborn baby, and their health and development beyond the neonatal period are well known. Specifically, there is compelling evidence in both full-term and preterm infant populations that breastfeeding or maternal milk feeding benefits child neurodevelopment.^1^ Recently, there has been growing interest in the neurotherapeutic potential of intranasal administration of human breast milk for preterm brain injury acquired in the neonatal period.^2–4^

Keller et al. in 2018 first reported a small case-control series of preterm infants with severe intraventricular hemorrhage who received nasal drops of fresh breast milk daily (2 × 0.1 mL three to eight times a day) with informed parental consent for at least 28 days (in one case up to 105 days). A trend towards a lower incidence of severe porencephaly, progressive ventricular dilatation, and surgery for post-hemorrhagic hydrocephalus was noted in the intranasal breast milk group in this non-randomised open-label, pilot study.^3^ The transfer of neurotropic factors and stem cells derived from human breast milk had been hypothesized as the mechanism responsible for the potential neuroprotective benefit. In 2022, Gallipoli and colleagues reported on their small pilot safety and feasibility study done in Canada at the Pediatric Academic Society meeting in Denver, CO.^4^ Intranasal milk was administered to 37 preterm infants with a median gestation of 27 weeks, and median birth weight of 1000 grams. They showed that these preterm infants were able to tolerate nasal milk therapy through 28 days of life without major safety events. Detailed results are not yet available, and the group is planning a larger trial.

The intranasal route has been touted as a potential access for neurotherapeutics in adult and pediatric brain conditions for a number of years.^5^ The main reason being the blood brain barrier is not easily accessed by many cells, biologicals, synthetic materials and molecules when they are given systemically or intravenously to patients. Intrathecal and direct injections into brain spaces are considered too invasive, and hence the intranasal route may serve as a good direct access to the brain. Nasal vascularity and the permeable neonatal blood brain barrier may potentially allow stem cell and other molecule delivery to brain tissue. Stem cells and cell therapies are the new frontier in neonatal medicine, with a number of cell types in pre-clinical and clinical trials currently.^6^ The intranasal route has been studied for a number of stem cell and cell therapy applications in pre-clinical models of neonatal brain injury. These include intranasal mesenchymal stromal cells,^7,8^ amnion epithelial cells,^9^ and neural stem cells.^10^ To consider administering the stem cells and the neurotrophic growth factors present in breast milk though the intranasal route is fascinating.

In this issue of *Pediatric Research*, Kaps and colleagues report on their in-vitro study of human preterm colostrum for regeneration of neurite outgrowths in murine olfactory bulb explants.^11^ This was a proof of concept, pilot in vitro study following the group’s earlier human study mentioned above,^3^ where they proposed that preterm milk, especially preterm colostrum might be the richest source of neurotrophic factors. To test this hypothesis, milk was collected at the colostrum stage and mature age from five different individuals who had given birth to preterm babies, and pooled milk used for the laboratory experiments. Mice olfactory explants were co-cultured with pooled preterm colostrum, pooled mature milk or medium with no supplements, and neurite outgrowths studied using a stereo microscope. Both types of milk (preterm colostrum and mature) increased neurite outgrowth, though this was more evident with preterm colostrum. There is a fascinating video of radial cell migration in the neurite outgrowth in a supplementary file of the paper. Milk proteomics using mass spectrometry and gene ontology enrichment studies were also conducted, which showed differences in important genes regulating neuroactive proteins in the preterm colostrum as compared to mature milk. These included proteins involved in neuron axon guidance, neuromodulation and cell adhesion.

Animal studies using intranasal administration of growth factors have raised interest in breast milk’s potential to improve neural injury.^12^ Colostrum is known to have significantly higher numbers of immunoglobulins, antibodies, growth factors and other proteins. Kim et al investigated the anti-apoptotic effects of bovine colostrum using organotypic hippocampal slice cultures and an intracerebral hemorrhage animal model.^13^ They found that colostrum treatment significantly suppressed hemorrhage induced cell death. Bovine colostrum has also been to shown have anti-oxidant properties in a bilateral common carotid artery occlusion-induced global ischemia-reperfusion injury model.^14^ Recently, some neuroprotection has also been reported with goat’s milk.^15^ Understanding the mechanisms of neuroprotection seen in these studies is really important, knowing that generally a significant publication bias exists for pre-clinical literature with predominantly positive studies reported. Replicating and reproducing the results seen in this current study by Kaps et al is critical hence to further elucidate the composition of colostrum and breast milk, and possible neurological benefits which may be seen with intranasal administration of human breast milk.

On balance, this is a very exciting field of development, and offers a potentially promising and practical benefit of human breast milk, which needs to be tested in larger pre-clinical and clinical studies.
